# Dual Tracer 68Ga-DOTATOC and 18F-FDG PET Improve Preoperative Evaluation of Aggressiveness in Resectable Pancreatic Neuroendocrine Neoplasms

**DOI:** 10.3390/diagnostics11020192

**Published:** 2021-01-28

**Authors:** Paola Mapelli, Stefano Partelli, Matteo Salgarello, Joniada Doraku, Francesca Muffatti, Marco Schiavo Lena, Stefano Pasetto, Carolina Bezzi, Valentino Bettinardi, Valentina Andreasi, Paola Maria Vittoria Rancoita, Luigi Gianolli, Maria Picchio, Massimo Falconi

**Affiliations:** 1 Vita-Salute San Raffaele University, Via Olgettina 58, 20132 Milan, Italy; mapelli.paola@hsr.it (P.M.); partelli.stefano@hsr.it (S.P.); bezzi.carolina@hsr.it (C.B.); andreasi.valentina@hsr.it (V.A.); falconi.massimo@hsr.it (M.F.); 2 Nuclear Medicine Department, IRCCS San Raffaele Scientific Institute, Via Olgettina 60, 20132 Milan, Italy; bettinardi.valentino@hsr.it (V.B.); gianolli.luigi@hsr.it (L.G.); 3 Pancreatic Surgery Unit, Pancreas Translational & Clinical Research Centre, IRCCS San Raffaele Scientific Institute, Via Olgettina 60, 20132 Milan, Italy; muffatti.francesca@hsr.it; 4 Department of Nuclear Medicine, Sacro Cuore Don Calabria Hospital, Viale Luigi Rizzardi, 4, 37024 Negrar, Italy; matteo.salgarello@sacrocuore.it (M.S.); joniada.doraku@sacrocuore.it (J.D.); stefano.pasetto@sacrocuore.it (S.P.); 5 Pathology Department, IRCCS San Raffaele Scientific Institute, Via Olgettina 60, 20132 Milan, Italy; schiavolena.marco@hsr.it; 6 University Centre of Statistics in the Biomedical Sciences, Vita-Salute San Raffaele University, Via Olgettina 58, 20132 Milan, Italy; rancoita.paolamaria@unisr.it

**Keywords:** pancreatic neuroendocrine neoplasms, NEN, 68Ga-DOTA, 18F-FDG, PET/CT, dual tracer, prognostic value, risk stratification, surgery, resection, staging

## Abstract

Purpose: To define an imaging risk profile in a population of patients affected by Pancreatic neuroendocrine neoplasms (PanNENs) candidates to surgery, by assessing the predictive role of 68Ga-DOTATOC and 18F-FDG PET/CT and PET/MR derived parameters in risk stratification, particularly regarding histological features of aggressive behaviour. Patients and methods: Retrospective study including 83 patients (53 males, 30 females; median age: 60 years, interquartile range 52–66.5), who underwent to 68Ga-DOTATOC (PET/CT: *n* = 77; PET/MR: *n* = 6) and, 68/83 patients, also to 18F-FDG PET (PET/CT: *n* = 65; PET/MR: *n* = 3) before surgery for PanNEN between 2011 and 2019, with available histological and follow-up data. The PET scans were interpreted with both qualitative (positive vs. negative) and semiquantitative measurements as follows: maximum and mean standardized uptake value (SUVmax and SUVmean) for both 18F-FDG and 68Ga-DOTATOC scans, metabolic tumour volume (MTV) and tumour lesion glycolysis (TLG) for 18F-FDG scans and somatostatin receptor density (SRD) and total lesion somatostatin receptor density (TLSRD) for 68Ga-DOTATOC PET. Receiver Operating Characteristics (ROC) curve analysis was used to investigate the performance of several PET parameters in predicting tumour stage or characteristic. For each PET parameter, the optimal cut-off was derived. Logistic regression analysis was used to assess if the PET parameters, categorized with the optimal cut-off values, were able to predict significantly the corresponding tumour stage or characteristic. Results: Overall, 29 (35%) patients had G1, 49 (59%) a G2 and five (6%) had a G3 PanNEN. The median Ki-67 index was 4% (interquartile range: 1–8%). SRD and TLSRD significantly discriminated between pT3 or pT4 PanNEN versus pT1 or pT2, as well as 18F-FDG MTV and TLG. 68Ga-DOTATOC SUVmax was able to significantly predict the presence of distant metastases with a threshold of 51.27 (sensitivity and specificity of 85.7 and 68.1%, respectively). 18F-FDG MTV and TLG were predictors of angioinvasion. The cut-off threshold for MTV was 7.98 (sensitivity and specificity of 69.7 and 82.4%, respectively) (*p* = 0.0004) whereas the cut-off for TLG was 32.4 (sensitivity and specificity of 69.7% and 82.4%, respectively) (*p* = 0.0004). Conclusion: Dual tracer 68Ga-DOTATOC and 18F-FDG PET scans provide relevant information regarding tumour behaviour and aggressiveness, implementing the diagnostic preoperative work-up.

## 1. Introduction

Pancreatic neuroendocrine neoplasms (PanNENs) represent a heterogeneous group of neoplasia, with a wide spectrum of clinical presentations and aggressiveness. Although the overall prognosis is usually better when compared to other solid tumours, several factors may influence patients’ outcome, including grade and extent of disease [1,2].

The World Health Organization (WHO) classification of Tumours of Endocrine Organs 2017 and WHO Classification of Tumours, Digestive System Tumours, 2019 [1,2] differentiate PanNENs with a well differentiated morphology (PanNETs: pancreatic neuroendocrine tumours) according to Ki67 proliferative index and mitotic count, which might also have impact on patients’ survival [3].

Surgical resection is the first-line therapy for localized PanNENs as well as for advanced, but still resectable neoplasms [4,5,6].

Several imaging modalities are currently used to define morphological and functional features of PanNENs and to stage the disease [7]. Regarding morphological imaging, Computed Tomography (CT) and Magnetic Resonance Imaging (MRI) are both essential to verify the resectability of the pancreatic lesion. However, these modalities are known to have limitations regarding the definition of pathological lymph nodes, which is based on the short axis diameter. In the field of molecular imaging, 68Ga-DOTA-peptides PET/CT seems to be the most accurate imaging modality to define somatostatin receptors (SSTR) expression during staging and restaging of patients with PanNENs [8].

Another radiotracer that is mainly used to stage and restage PanNET and pancreatic neuroendocrine carcinoma (PanNEC) is 18F-Fluorodeoxyglucose (18F-FDG) [9,10].

The combined role of DOTA-peptides PET/CT and 18F-FDG PET/CT has been investigated in PanNENs mainly focusing on their diagnostic accuracy [11]. In fact, only limited data have been reported regarding the prognostic value of these imaging modalities in PanNENs risk stratification, often including patients with NENs of different origin and at different stages of disease (i.e., primary observation, recurrence; metastatic, non-metastatic) [7,12,13,14].

Even less evidence is available on the role of a dual tracer approach in patients who are candidates to surgery for PanNENs, aiming to define possible imaging-derived parameters able to describe preoperatively a more aggressive phenotype.

In the present study, we sought to define an imaging risk profile in a population of patients affected by PanNEN candidates to surgery, by assessing the predictive role of 68Ga-DOTATOC and 18F-FDG PET/CT or PET/MR derived parameters in risk stratification, particularly regarding nodal status and histological features of aggressive behaviour.

## 2. Materials and Methods

### 2.1. Patients

This retrospective bicentric study included 83 patients (53 males, 30 females; median age: 60 years, interquartile range 52–66.5). In particular, 60/83 patients were studied at the Department of Nuclear Medicine, IRCCS Sacro Cuore Don Calabria Hospital (Negrar, Verona) and 23/83 at the Department of Nuclear Medicine at IRCCS San Raffaele Hospital (Milan).

All 83 patients underwent to 68Ga-DOTATOC and, 68/83 patients, also to 18F-FDG PET within one-month before surgery for PanNEN, between May 2013 and May 2019.

Additional inclusion criteria were availability of histological and follow-up data.

For all patients studied at IRCCS San Raffaele Scientific Institute, a signed informed consent form to undergo PET/CT and PET/MR scanning and for anonymous publication of disease related information was obtained. In addition, patients studied at IRCCS Sacro Cuore Don Calabria Hospital were included in the present study under the registration code 2155CESC provided by The Ethical Committee of Verona University. For one minor included in the study, signature of the informed consent form was obtained by both the minor and his parents or Legally Authorized Representatives.

### 2.2. Imaging Acquisition

Regarding the PET/CT scans performed at the Department of Nuclear Medicine, IRCCS Sacro Cuore Don Calabria Hospital, the studies were acquired on a mCT 64-slice scanner (mCT Biograph and Biograph mCT Flow, Siemens, Munich, Germany, *n* = 60). Patients fasted for at least 6 h before the examinations. Patients with a blood glucose level higher than 150 mg/dL at the time of PET/CT scans had the procedures postponed. Images from the vertex to the proximal femur were obtained with patients in supine position. 68Ga-DOTATOC and 18F-FDG PET/CT were performed on the same day, with 68Ga-DOTATOC PET/CT performed prior to 18F-FDG scan with a time interval of 6 h between the two scans. Each PET/CT scan was acquired approximately 1 h after intravenous injection of the tracer (68Ga-DOTATOC: 1.5 MBq/kg; 18F-FDG: 2.96 MBq/kg). The duration of bed position was 3 and 2 min for 68Ga-DOTATOC PET/CT and 18F-FDG PET/CT, respectively. The acquisition time of both PET/CT scans also accounted for Body Mass Index (BMI), with 30 additional seconds of acquisition in patients with BMI > 28 and 1 additional minute for patients with BMI > 30. CT images were obtained with the use of a standardized protocol of 120 kV, care dose of 100 mAs, tube rotation time of 0.5 s per rotation, a pitch of 1.4, and a slice thickness of 5 mm. Attenuation corrected PET/CT fusion images were reviewed in 3 plans (transaxial, coronal, and sagittal) with True-D software (Siemens).

Regarding the PET scans acquired at IRCCS San Raffaele Hospital, studies were acquired using two PET/CT (Discovery-STE, *n* = 10, and Discovery-690, *n* = 7) and one PET/MR (Signa PET/MRI, General Electric Medical Systems, Wakesha, WI, USA) scanner (*n* = 6). Patients’ preparation was performed following the same protocol as described above, with 68Ga-DOTATOC and 18F-FDG PET scans performed in two different days but within one week one from the other (68Ga-DOTATOC: 2.2–2.5 MBq/kg; 18F-FDG: 4.7 MBq/kg).

PET/CT acquisition protocols consisted in a low dose whole body CT scan (kVp = 120, AutomA (30–150 mA) for anatomical localization followed by a whole body PET study (2.5/field of view-FOV) for 18F-FDG and a whole body PET study (3 min/FOV) for 68Ga DOTATOC.

PET/MR acquisition protocol consisted in an axial MR-based Attenuation Correction (MRAC) sequence (Dixon based), to be used for MR based attenuation correction of PET data, an axial LAVA Flex sequence, to be used for anatomical localization followed by a whole body PET study (4 min/FOV) simultaneously acquired to the MR data for both 18F-FDG and 68Ga-DOTATOC.

Despite the use of different tomographs between the two Centres, the performances of PET scanners are well comparable. In fact, the PET/CT mCT system, the PET/CT Discovery 690 and the SIGNA PET/MR all belong to the new-generation tomographs, incorporating the same type of detector and advanced reconstruction techniques such, fully 3D ordered subset expectation-maximization (OSEM) algorithm, time-of-flight (TOF) and point-spread-function (PSF). Although PET/CT Discovery-STE belongs to a previous generation class of tomographs, also allows a fully 3D acquisition as well as a fully 3D reconstruction, thus providing very good clinical performances/images. Furthermore, all PET tomographs are well calibrated for an accurate detection of absolute radioactivity concentration. Moreover, the reconstruction parameters are as much as possible aligned, with the aim to obtain comparable clinical images, considering the specificity of each scanner, which does not allow the set-up of exactly the same parameters (e.g., number of subsets) even within the same vendor (e.g., General Electric Medical System - GEMS). In fact, the main reconstruction parameters were: mCT: 21 Subsets, 3 Iterations, Gaussian Post Filter FWHM 4 mm + TOF + PSF, Discovery 690: 18 Subsets, 3 Iterations, Gaussian Post Filter FWHM 4 mm + TOF + PSF, SIGNA PET/MR: 16 Subsets, 3 Iterations, Gaussian Post Filter FWHM 4 mm + TOF + PSF, Discovery STE: 28 Subsets, 2 Iterations, Gaussian Post Filter FWHM 4 mm.

### 2.3. PET/CT and PET/MR Image Analysis and Estimation of PET-Derived Parameters

The PET/CT and PET/MR scans where interpreted for both the institutions by two readers with more than 10 years of experience, aware of all clinical and imaging details, by means of both qualitative (positive vs. negative) and semiquantitative measurements. Findings with tracer uptake higher than physiological biodistribution were considered as positive.

For each scan, the pancreatic primary lesion has been specifically contoured and used for the following image analysis.

A volume of interest (VOI) defining the focal pathological uptake, corresponding to the primary tumour, was contoured on transaxial PET images both on 68Ga-DOTATOC and 18F-FDG PET scan. The applied segmentation was 3D, using a thresholding-based model with a cut off of 40% of the maximum standardized uptake value (SUVmax).

The following PET semiquatitative parameters have been assessed: standardized uptake value (SUV) max and mean for both 68Ga-DOTATOC and 18F-FDG PET, somatostatin receptor density (SRD) and total lesion somatostatin receptor density (TLSRD) for 68Ga-DOTATOC PET scans, metabolic tumour volume (MTV) and tumour lesion glycolysis (TLG) for 18F-FDG PET scans.

### 2.4. Surgery

Surgical resection was planned according to the site of the tumour and its dimension. Atypical resections, including middle pancreatectomy (MP) and enucleation, were performed in the presence of PanNEN less than or equal to 2 cm in size. PanNEN ≤ 2 cm with a strict relationship with the main pancreatic duct (MPD) were excluded from enucleation. Typical resection included pancreaticoduodenectomy (PD), distal pancreatectomy (DP) with or without splenectomy and total pancreatectomy. In the presence of preoperative high-risk features of recurrence (i.e., large diameter, vascular or nearby organs infiltration, presence of liver metastases) selected patients (*n* = 5) were submitted to preoperative neoadjuvant treatment (2/5 peptide-receptor radionuclide therapy—PRRT, 1/5 octreotide analogues, 2/5 combined chemotherapy and octreotide analogues). Overall, 14 patients had liver metastases. In the presence of multiple, non-resectable, liver metastases, a palliative primary resection was carried out (*n* = 4) whereas in the remaining 10 patients a radical resection (R0, *n* = 9, R1, *n* = 1) was performed.

### 2.5. Pathology

All patients included in this study underwent surgery for localized or metastatic disease. The Ki67 evaluation was expressed as a percentage based on the count of Ki67-positive cells within the tumour, using NCL-L-Ki67-MM1 (Novocastra, Newcastle Upon Tyne, UK) and KI67 CL. 30-9 (Ventana Medical System Inc., Tucson, ARI, USA), antibodies; the spot with the highest immunostaining was considered when intratumoural heterogeneity was present. The 2017 WHO classification was applied to determine tumour grade, and tumours were classified as PanNET G1 (Ki67 < 3%) or PanNET-G2 (Ki67 3–20%) or PanNEN-G3 (Ki-67 > 20%). Patients with poorly differentiated lesions with Ki67 > 20% were classified as PanNEC.

### 2.6. Statistical Analysis

The Receiver Operating Characteristics (ROC) curve analysis was used to investigate the performance of several PET parameters in predicting tumour status or characteristics. Area under the ROC Curve (AUC) were interpreted as: 0.7 ≤ AUC < 0.8 acceptable, 0.8 ≤ AUC < 0.9 excellent, ≥0.9 outstanding. For each PET parameter, the optimal cut-off was derived using the standard method, consisting in choosing that value corresponding to the point on the ROC curve nearest to the upper left corner of the ROC graph. Logistic regression analysis was then used to assess whether the PET parameters categorized with the optimal cut-off could significantly predict the corresponding tumour status or characteristic. For each tumour characteristic, *p*-values were adjusted with Bonferroni’s correction for accounting for multiple testing considering all PET parameters. Descriptive statistics of time to event outcomes were computed by using the Kaplan–Meier estimator. *p*-Values less than 0.05 were considered significant. Statistical analyses were performed using R 3.5.0 (http://www.R-project.org/).

## 3. Results

### 3.1. Patients’ Population

All 83 patients underwent to 68Ga-DOTATOC PET and, in 68/83 patients, 18F-FDG PET was also performed (6/83 patients underwent PET/MR and 77/83 underwent PET/CT). Overall, 78 (94%) patients underwent a resection with curative intent (R0–R1), whereas a R2-resection was performed in 5 cases (6%).

According to the 2017 WHO classification [2], 29 (35%) patients had G1, 49 (59%) a G2 and 5 (6%) had a G3 (*n* = 4 PanNET-G3, *n* = 1 PanNEC-G3) PanNEN. The median Ki-67 index was 4% (interquartile range: 1–8%). One out of 83 patients had a functioning tumour (insulinoma), 14/83 (16.9%) presented distant metastases (7/14 at the time of diagnosis; 7/14 developed during follow-up), 31/78 (39.7%) had nodal metastases and 40/82 (48.8%) showed angioinvasion at histological examination. Patients’ characteristics are reported in Table 1.

### 3.2. 68Ga-DOTATOC PET-Derived Parameters as Predictors for Clinicopathological Features of Primary Tumour

68Ga-DOTATOC PET was positive in correspondence of the primary tumour in all the patients included in the study. In 9/83 patients 68Ga-DOTATOC PET was positive in correspondence of distant metastases (9/9 liver).

Descriptive statistics of 68Ga-DOTATOC PET-derived parameters are reported in Table 1.

Table 2 and Table 3 show the results of ROC curve analysis. SRD and TLSRD were the only parameters significantly associated with larger tumours (pT3 or pT4 PanNEN versus pT1 or pT2), showing an optimal (AUC = 0.8177, *p* < 0.0001) and acceptable AUC (AUC = 0.7751, *p* = 0.0002), respectively. The optimal cut-off values were found to be 18.34 (*p* = 0.0002) for SRD and 275.41 (*p* = 0.0031) for TLSRD, with correspondent sensitivity and specificity of 78.1 and 72.5% for SRD, and 75 and 66.7% for TLSRD.

Only SUVmax was the only parameter significantly associated with the presence of distant metastases (AUC = 0.7474, *p* = 0.0298), with an optimal threshold of 51.27 (*p* = 0.0124), achieving correspondent sensitivity and specificity of 85.7 and 68.1%, respectively (Table 3).

### 3.3. 18F-FDG PET-Derived Parameters as Predictors for Clinicopathological Features of Primary Tumour

18F-FDG PET/CT showed uptake in correspondence of the primary tumour in 46/68 patients. In 6/83 patients 18F-FDG PET/CT was positive in correspondence of distant metastases (6/6 liver).

**Figure 1:**
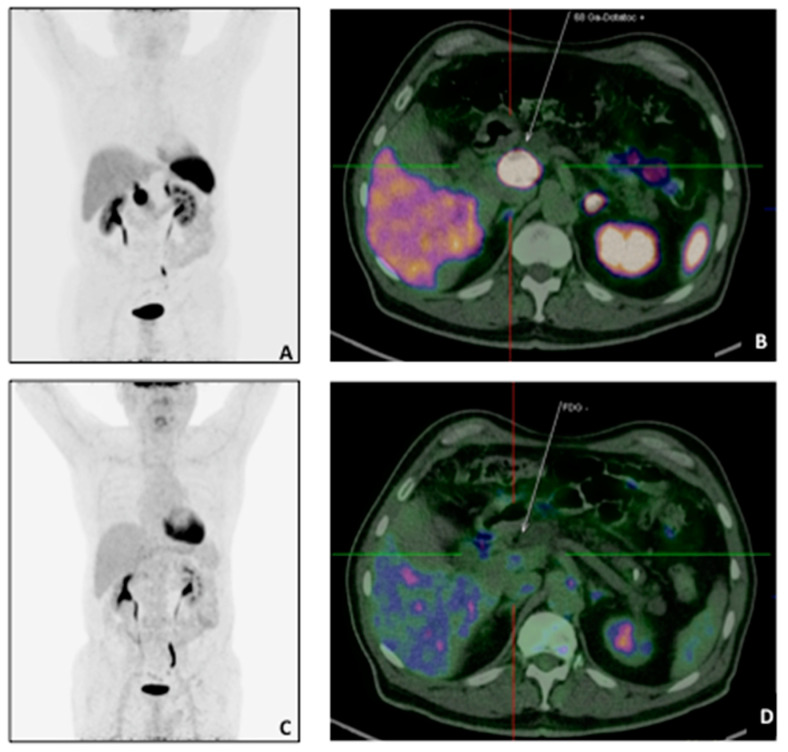
A 73 years-old patient with pancreatic NET (G2, pT2N1M0) underwent 68Ga-DOTATATE and 18F-FDG PET/CT for staging purpose. The68Ga-DOTATATE MIP (**A**) and transaxial sections (**B**) showed intense uptake in correspondence of the primary tumour (white arrow: SUVmax: 107.1) with a negative 18F-FDG scan (MIP: (**C**); transaxial: (**D**).

Among the 22 patients with a negative scan 12/22 (54.5%) had G1 (Ki67: 0.6% in 1/12, 1% in 10/12, 2% in 1/12) and 10/22 (45.4%) with G2 (Ki67: 3% in 1/10; 4% in 1/10, 5% in 3/10, 10% in 2/10, 17% in 1/10, 18% in 1/10, and 20% in 1/10) (Figure 1).

Descriptive statistics of 18F-FDG PET-derived parameters are reported in Table 1.

**Figure 2:**
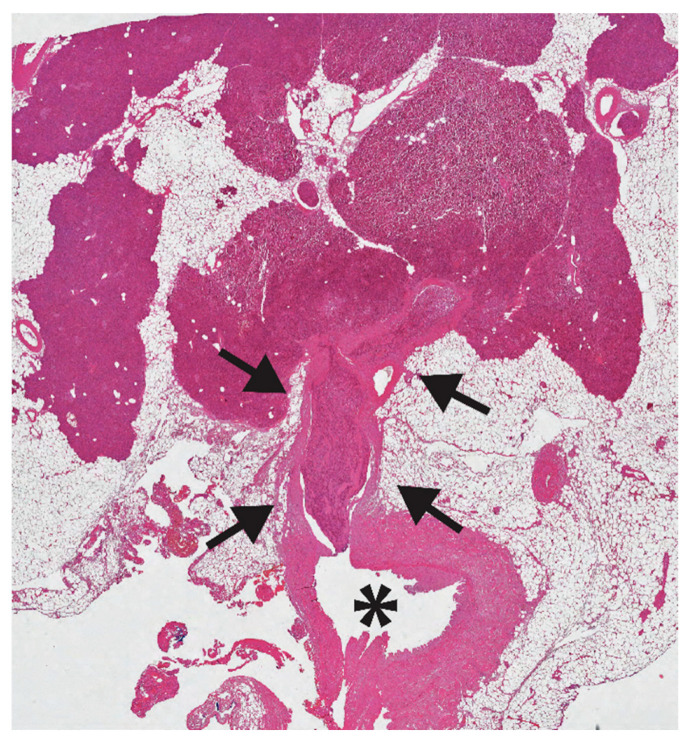
A huge neoplastic thrombus is seen (black arrows) in a branch of the splenic vein (asterisk). This 43-years old man underwent distal pancreatectomy for a 2.3 cm panNET, Ki67 3%, with loss of Death Domain Associated Protein (DAXX) nuclear expression (hematoxylin eosin stain; 20×).

**Figure 3:**
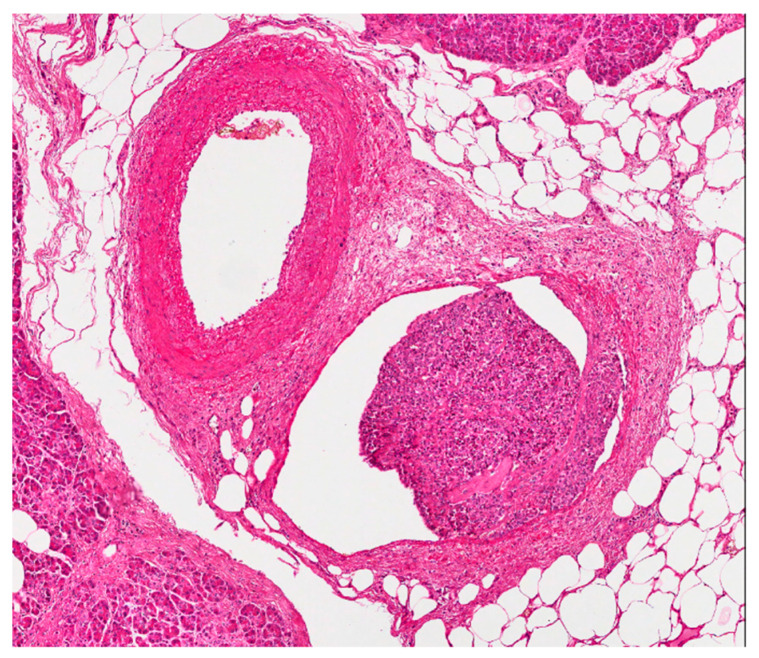
In paired vessels, neoplastic thrombus is seen in the venula (in the middle of the picture). The patient is the same of Figure 2 (hematoxylin eosin stain; 100×).

Table 4 and Table 5 show the results of ROC curve analysis. MTV and TLG were significantly associated with tumour size, both with an acceptable AUC (MTV: AUV = 0.7498, *p* = 0.0029; TLG: AUC = 0.7392, *p* = 0.0051). The cut-off thresholds for MTV was 17.36 (*p* = 0.0005) and correspondent sensitivity and specificity were 58.2 and 94.6% (*p* = 0.0005), respectively; the cut-off threshold for TLG was 32.4 (*p* = 0.0116), with correspondent sensitivity and specificity of 65.5 and 74.4% (*p* = 0.0116), respectively. MTV and TLG were able to predict the presence of angioinvasion, with an acceptable AUC (for MTV: AUC = 0.7656, *p*-value = 0.0011; for TLG: AUC = 0.7603, *p* = 0.0015); cut-off thresholds, sensitivity and specificity were 7.98, 69.7, 82.4% (*p* = 0.0004) for MTV and 32.4, 69.7, 82.4% (*p* = 0.0004) for TLG (Figure 2 and Figure 3; Table 4 and Table 5). All parameters were able to significantly predict Ki-67 ≥ 3% vs. <3% with an acceptable AUC (Table 4), but the corresponding categorized variables obtained with the optimal cut-offs resulted to achieve a *p*-value slightly above the significance level (Table 5).

### 3.4. Follow-Up

Mean follow-up was 22.8 months (95% confidence interval, CI: 19.1–26.6 months), with a median DFS of 52.9 months (95% CI: 47.1–58.6).

At last follow-up, 82 patients were alive, 75/82 patients (91.5%) were free of disease, 1/82 patient had (1.2%) a disease in regression, 3/82 (3.7%) patients had stable disease and 3/82 (3.7%) had a progressive disease. The only patient who died because of PanNEN was a 38 year-old female with G2 PanNET (Ki67 = 15%; pT4N0M1).

## 4. Discussion

The availability of an accurate risk stratification assessment in patients affected by patients with PanNENs who are candidates to surgery is of utmost importance.

In the present study, the impact of combined 68Ga-DOTATOC and 18F-FDG PET in providing a risk profile of PanNENs behaviour prior to surgery, particularly regarding possible aggressive behaviour, has been investigated.

The complementary role of these two imaging modalities has been reported in some studies that analysed patients affected by NENs of different origins and treated with different approaches according to tumour stage [13,14].

This complementarity supports the hypothesis that the use of dual tracer approach may resolve the limitations linked to histopathologic grading and its heterogeneity. [14]

Cingarlini et al. assessed the role of combined 68Ga-DOTATATE and 18F-FDG PET/CT in PanNENs and tested the correlation between imaging positivity and tumour grade suggesting a high positive predictive value of 18F-FDG PET/CT in identifying G2 forms. [15] However, they did not analyse the possible role of combined 68Ga-DOTATATE and 18F-FDG PET/CT in risk stratification in a selected cohort of patients who underwent surgery, as it is has been done in the present paper. Recently, Abdulrezzak et al. investigated the additional contribution of combined 68Ga-DOTATATE and 18F-FDG PET/CT in NENs and described the complementary role of these imaging modalities in treatment approach and response assessment, suggesting the reliability of semiquantitative parameters, such as SUV, to evaluate prognosis and tumour aggressiveness [12]. The present study presents several differences compared to the one of Abdulrezzak et al. First, our cohort of patients is homogenous in terms of site and treatment strategy; in fact, the value of the present study is that patients affected by pancreatic NEN and candidate to surgery are included (being all histological data available), focusing on risk predictors.

Only few studies investigated the possible prognostic role of 68Ga-DOTA peptide PET in defining a risk definition before treatment.

Campana and colleagues, in a cohort of 47 patients with NENs, firstly demonstrated the correlation between SUVmax and clinical and pathologic features, being these parameters also an accurate prognostic index [16]. However, also in their study, patients were heterogeneous in terms of site of NEN origin and treatment approach, making difficult to define dual tracers PET value in resectable PanNEN.

Lately, Ambrosini et al. demonstrated for the first time the relevance of SUVmax as a prognostic factor in patients with G1 and G2 PanNENs at different times of disease presentation. However, once again, patients included were candidates to different treatment strategies, despite a possible relevance of SUVmax in improving disease characterization and management was clearly suggested [7].

A meaningful result from the present study relies on the predictive power of 68Ga-DOTATATE SUVmax in predicting metastatic involvement with a sensitivity and specificity of 85.7 and 68.1%, respectively, also identifying a cut-off value of 51.27. Another aspect that should be stressed is that 18F-FDG MTV and TLG were predictive for angioinvasion. This feature of aggressiveness, currently evincible only from histological examination, could be predicted potentially by 18F-FDG PET/CT with high values of sensitivity and specificity.

Recently, Toriihara and colleagues demonstrated the prognostic value of the sum of SRD and TLSRD of all detected lesions in patients affected by well-differentiated NET [17]. The population of this study included primary NETs of different origins, with patients undergoing different treatments according to disease stage (surgical, medical, PRRT) and the possible prognostic value of 68Ga-DOTATATE PET/CT on survival, was reported. Authors underlined the relevance of considering also 18F-FDG parameters for NETs characterization, especially in those patients presenting NET with less affinity for 68Ga-DOTA peptides and higher uptake of 18F-FDG. However, in their patients’ cohort, only 68Ga-DOTATATE scans have been performed. Conversely, in the present paper, the complementarity of the dual tracer approach, by considering the simultaneous contribution of both 68Ga-DOTATOC and 18F-FDG parameters in predicting tumour behaviour, has been reported.

In view of these results, if confirmed, the complementary role of 68Ga-DOTATOC and 18F-FDG PET/CT could be suggested in the preoperative setting of PanNENs, changing, subsequently, the diagnostic algorithm [18]. In the present study, some limitations should be pointed out beyond the retrospective nature of the study. In total, 15 patients only underwent 68-Ga DOTATOC PET/CT and not 18F-FDG PET/CT and this might have slightly reduced the statistical significance of the analysis. Patients underwent PET scans at different Institutions and on different scanners; however, the applied acquisitions and reconstruction protocols made the obtained semiquantitative values reproducible and comparable. The population size could be also a matter of concern. However, it represents one of the largest series of patients affected by PanNEN investigated with dual tracer modality in the preoperative setting.

## 5. Conclusions

Dual tracer PET/CT, by means of using 68Ga-DOTA-peptides and 18F-FDG as radiotracers, provides relevant information regarding tumour behaviour and aggressiveness also in patients eligible to pancreatic resection according to the current usual indications. The addition of this tool seems to provide a better delineation of disease aggressiveness, therefore favouring a more tailored surgical approach.

## Figures and Tables

**Table 1:** Main characteristics of the patients and the underlying disease.

Number of Patients	83
**Male, *n* (%)**	53 (63.9)
**Female, *n* (%)**	30 (36.1)
**Median age, years (IQR)**	60 (52–66.5)
**Pancreatic site, *n* (%)**	
Head	31 (37.4)
Isthmus	4 (4.8)
Body	26 (31.3)
Tail	20 (24.1)
Multifocal	1 (1.2)
Uncinate process	1 (1.2)
**Median Lesion diameter, mm (IQR)**	28 (20–35.5)
**Median Ki-67, % (IQR)**	4 (1–8)
**WHO 2017 Classification** [1], ***n* (%)**	
G1	29 (35.0)
G2	49 (59.0)
G3	5 (6.0)
**TNM** [4]	
**T** (*n*, %)	16 (19.3)
T1	35 (42.2)
T2	30 (36.1)
T3	2 (2.4)
T4	
**N** (*n*, %)	
N0	47 (56.6)
N1	31 (37.4)
NX	5 (6.0)
**M** (*n*, %)	
M0	69 (83.1)
M1	14 (16.9)
**68Ga-DOTATOC PET parameters, median (IQR)**	
SUVvmax	56.70 (33.04–80.66)
SUVmean	18.97 (12.90–35.23)
SRD	16.11 (6.16–43.95)
TLSRD	271.2 (121.0–917.0)
**18F-FDG PET parameters, median (IQR)**	
SUVmax	5.45 (0–8.03)
SUVmean	3.28 (0–3.97)
MTV	5.65 (0–20.15)
TLG	19.20 (0–89.64)

IQR: interquartile range; SUV: standardized uptake value; MTV: metabolic tumour volume; TLG: total lesion glycolysis; SRD: somatostatin receptor density; TLSRD: total lesion somatostatin receptor density.

**Table 2:** 68Ga-DOTATOC PET-derived parameters as predictors for clinicopathological features of PanNENs. In the analysis of each clinicopathological feature, *p*-values were adjusted considering both 68Ga-DOTATOC and 18F-FDG PET-derived parameters.

Clinicopathological Features	SUVmax		SUVmean		SRD		TLSRD	
	AUC	*p*-Value	AUC	*p*-Value	AUC	*p*-Value	AUC	*p*-Value
pT3-T4 vs. pT1-T2	0.5260	1.0000	0.5490	1.0000	0.8177	**<0.0001**	0.7751	**0.0002**
Ki-67 ≥ 3% vs. <3%	0.5105	1.0000	0.5016	1.0000	0.6232	0.5278	0.6086	0.8437
Angioinvasion	0.5188	1.0000	0.6253	0.4112	0.5315	1.0000	0.6036	0.8622
Distant Metastases	0.7474	**0.0298**	0.6801	0.2789	0.5823	1.0000	0.5052	1.0000
Lymph nodal metastases	0.5103	1.0000	0.6050	0.9556	0.5093	1.0000	0.5374	1.0000

**Table 3:** Identification of the threshold of 68Ga-DOTATOC PET-derived parameters for predicting clinicopathological features of PanNENs. In the analysis of each clinicopathological feature, *p*-values were adjusted considering both 68Ga-DOTATOC and 18F-FDG PET-derived parameters.

Clinicopathological Features	SUVmax				SUVmean				SRD				TLSRD			
	Thr	Sens	Spec	*p*-Value	Thr	Sens	Spec	*p*-Value	Thr	Sens	Spec	*p*-Value	Thr	Sens	Spec	*p*-Value
pT3-T4 vs. pT1-T2	56.65	56.3%	52.9%	1.0000	22.00	68.8%	47.1%	1.0000	18.34	78.1%	72.5%	**0.0002**	275.41	75.0%	66.7%	**0.0031**
Ki-67 ≥ 3% vs. <3%	54.30	50.0%	62.1%	1.0000	16.25	64.8%	55.2%	0.6511	11.55	66.7%	55.2%	0.4505	174.69	75.9%	48.3%	0.2188
Angioinvasion	61.92	47.5%	59.5%	1.0000	16.25	72.5%	54.8%	0.1094	20.40	50.0%	59.5%	1.0000	217.12	72.5%	50.0%	0.3123
Distant Metastases	51.27	85.7%	68.1%	**0.0124**	19.03	71.4%	53.6%	0.7731	21.00	64.3%	43.5%	1.0000	287.55	64.3%	50.7%	1.0000
Lymph nodal metastases	57.85	54.8%	53.2%	1.0000	18.05	71.0%	57.4%	0.1245	10.90	45.2%	68.1%	1.0000	266.70	58.1%	51.1%	1.0000

Thr: Threshold; Sens: Sensitivity (%); Spec: Specificity (%); SUV: standardized uptake value; SRD: somatostatin receptor density; TLSRD: total lesion somatostatin receptor density.

**Table 4:** 18F-FDG PET-derived parameters as predictors for clinicopathological features of PanNENs. In the analysis of each clinicopathological feature, *p*-values were adjusted considering both 68Ga-DOTATOC and 18F-FDG PET-derived parameters.

Clinicopathological Features	SUVmax		SUVmean		MTV		TLG	
	AUC	*p*-Value	AUC	*p*-Value	AUC	*p*-Value	AUC	*p*-Value
pT3-T4 vs. pT1-T2	0.6446	0.3140	0.6278	0.5483	0.7498	**0.0029**	0.7392	**0.0051**
Ki-67 ≥ 3% vs. <3%	0.7100	**0.0367**	0.7100	**0.0367**	0.7031	**0.0490**	0.7134	**0.0317**
Angioinvasion	0.6809	0.0762	0.6787	0.0835	0.7656	**0.0011**	0.7603	**0.0015**
Distant Metastases	0.6168	1.0000	0.5951	1.0000	0.6504	0.7101	0.6476	0.7591
Lymph nodal metastases	0.5029	1.0000	0.5068	1.0000	0.5777	1.0000	0.5729	1.0000

**Table 5:** Identification of the threshold of 18F-FDG PET-derived parameters for predicting clinicopathological features of PanNENs. In the analysis of each clinicopathological feature, *p*-values were adjusted considering both 68Ga-DOTATOC and 18F-FDG PET-derived parameters.

Clinicopathological Features	SUVmax				SUVmean				MTV				TLG			
	Thr	Sens	Spec	*p*-Value	Thr	Sens	Spec	*p*-Value	Thr	Sens	Spec	*p*-Value	Thr	Sens	Spec	*p*-Value
pT3-T4 vs. pT1-T2	6.20	55.2%	66.7%	0.5947	3.45	55.2%	69.2%	0.3654	17.36	58.6%	94.9%	**0.0005**	32.40	65.5%	74.4%	**0.0116**
Ki-67 ≥ 3% vs. <3%	4.90	71.7%	63.6%	0.0545	2.67	71.7%	63.6%	0.0545	4.25	67.4%	68.2%	0.0590	12.95	71.7%	63.6%	0.0545
Angioinvasion	6.32	54.5%	73.5%	0.1703	3.45	54.5%	73.5%	0.1703	7.98	69.7%	82.4%	**0.0004**	32.40	69.7%	82.4%	**0.0004**
Distant Metastases	6.36	61.5%	65.5%	0.6522	3.45	61.5%	63.6%	0.8406	7.98	69.2%	63.6%	0.3099	59.50	53.8%	76.4%	0.3064
Lymph nodal metastases	5.22	50.0%	59.5%	1.0000	3.16	53.6%	59.5%	1.0000	7.05	60.7%	62.2%	0.5608	32.40	57.1%	64.9%	0.6385

Thr: Threshold; Sens: Sensitivity (%); Spec: Specificity (%); SUV: standardized uptake value; MTV: metabolic tumour volume; TLG: tumour lesion glycolysis.

## References

[1] Lloyd R.V., Osamura R.Y., Klöppel G., Rosai J. (2017). WHO Classification of Tumours of Endocrine Organs. WHO Classification of Tumours.

[2] Nagtegaal I.D., Odze R.D., Klimstra D., Paradis V., Rugge M., Schirmacher P., Washington K.M., Carneiro F., Cree I.A., WHO Classification of Tumours Editorial Board (2020). The 2019 WHO classification of tumours of the digestive system. Histopathology.

[3] Bu J., Youn S., Kwon W., Jang K.T., Han S., Han S., You Y., Heo J.S., Choi S.H., Choi D.W. (2018). Prognostic factors of non-functioning pancreatic neuroendocrine tumor revisited: The value of WHO 2010 classification. Ann. Hepato-Biliary Pancreat. Surg..

[4] Scarpa A., Mantovani W., Capelli P., Beghelli S., Boninsegna L., Bettini R., Panzuto F., Pederzoli P., Delle Fave G., Falconi M. (2010). Pancreatic endocrine tumors: Improved TNM staging and histopathological grading permit a clinically efficient prognostic stratification of patients. Mod. Pathol..

[5] Falconi M., Bartsch D.K., Eriksson B., Klöppel G., Lopes J.M., O’connor J.M., Salazar R., Taal B.G., Vullierme M.P., O’toole D. (2012). ENETS Consensus Guidelines for the management of patients with digestive neuroendocrine neoplasms of the digestive system: Well-differentiated pancreatic non-functioning tumors. Neuroeindocrinology.

[6] Partelli S., Gaujoux S., Boninsegna L., Cherif R., Crippa S., Couvelard A., Scarpa A., Ruszniewski P., Sauvanet A., Falconi M. (2013). Pattern and clinical predictors of lymph node involvement in nonfunctioning pancreatic neuroendocrine tumors (NF-PanNETs). JAMA Surg..

[7] Ambrosini V., Campana D., Polverari G., Peterle C., Diodato S., Ricci C., Allegri V., Casadei R., Tomassetti P., Fanti S. (2015). Prognostic Value of 68Ga-DOTANOC PET/CT SUVmax in Patients with Neuroendocrine Tumors of the Pancreas. J. Nucl. Med..

[8] Bauckneht M., Albano D., Annunziata S., Santo G., Guglielmo P., Frantellizzi V., Branca A., Ferrari C., Vento A., Mirabile A. (2020). Somatostatin Receptor PET/CT Imaging for the Detection and Staging of Pancreatic NET: A Systematic Review and Meta-Analysis. Diagnostics.

[9] Garin E., Le Jeune F., Devillers A., Cuggia M., de Lajarte-Thirouard A.S., Bouriel C., Boucher E., Raoul J.L. (2009). Predictive value of 18F-FDG PET and somatostatin receptor scintigraphy in patients with metastatic endocrine tumors. J. Nucl. Med..

[10] Kayani I., Bomanji J.B., Groves A., Conway G., Gacinovic S., Win T., Dickson J., Caplin M., Ell P.J. (2008). Functional imaging of neuroendocrine tumors with combined PET/CT using ^68^Ga-DOTATATE (DOTA-DPhe^1^,Tyr^3^-octreotate) and ^18^F-FDG. Cancer.

[11] Muffatti F., Partelli S., Cirocchi R., Andreasi V., Mapelli P., Picchio M., Gianolli L., Falconi M. (2019). Combined ^68^Gallium-DOTA-peptides and ^18^F-FDG PET in the Diagnostic Work-up of Neuroendocrine Neoplasms (NEN): A Systematic Review. Clin. Transl. Imaging.

[12] Abdulrezzak U., Kurt Y.K., Kula M., Tutus A. (2016). Combined imaging with ^68^Ga-DOTA-TATE and ^18^F-FDG PET/CT on the basis of volumetric parameters in neuroendocrine tumors. Nucl. Med. Commun..

[13] Naswa N., Sharma P., Gupta S.K., Karunanithi S., Reddy R.M., Patnecha M., Lata S., Kumar R., Malhotra A., Bal C. (2014). Dual tracer functional imaging of gastroenteropancreatic neuroendocrine tumors using ^68^Ga-DOTA-NOC PET-CT and ^18^F-FDG PET-CT: Competitive or complimentary?. Clin. Nucl. Med..

[14] Simsek D.H., Kuyumcu S., Turkmen C., Sanlı Y., Aykan F., Unal S., Adalet I. (2014). Can complementary ^68^Ga-DOTATATE and ^18^F-FDG PET/CT establish the missing link between histopathology and therapeutic approach in gastroenteropancreatic neuroendocrine tumors?. J. Nucl. Med..

[15] Cingarlini S., Ortolani S., Salgarello M., Butturini G., Malpaga A., Malfatti V., D’Onofrio M., Davì M.V., Vallerio P., Ruzzenente A. (2017). Role of Combined ^68^Ga-DOTATOC and ^18^F-FDG Positron Emission Tomography/Computed Tomography in the Diagnostic Workup of Pancreas Neuroendocrine Tumors: Implications for Managing Surgical Decisions. Pancreas.

[16] Campana D., Ambrosini V., Pezzilli R., Fanti S., Labate A.M., Santini D., Ceccarelli C., Nori F., Franchi R., Corinaldesi R. (2010). Standardized uptake values of (68)Ga-DOTANOC PET: A promising prognostic tool in neuroendocrine tumors. J. Nucl. Med..

[17] Toriihara A., Baratto L., Nobashi T., Park S., Hatami N., Davidzon G., Kunz P.L., Iagaru A. (2019). Prognostic value of somatostatin receptor expressing tumor volume calculated from (68)Ga-DOTATATE PET/CT in patients with well-differentiated neuroendocrine tumors. Eur. J. Nucl. Med. Mol. Imaging.

[18] Mapelli P., Partelli S., Salgarello M., Doraku J., Pasetto S., Rancoita P., Muffatti F., Bettinardi V., Presotto L., Andreasi V. (2020). Dual tracer ^68^Ga-DOTATOC and ^18^F-FDG PET/computed tomography radiomics in pancreatic neuroendocrine neoplasms: An endearing tool for preoperative risk assessment. Nucl. Med. Commun..

